# Dose-Response Associations of Internet Use Time and Internet Addiction With Depressive Symptoms Among Chinese Children and Adolescents: Cross-Sectional Study

**DOI:** 10.2196/53101

**Published:** 2024-09-23

**Authors:** Juanjuan Li, Weidi Sun, Zeyu Luo, Yi Liu, Xuanyin Huang, Denan Jiang, Shuting Li, Jia Meng, Fang Gu, Ronghua Zhang, Peige Song

**Affiliations:** 1 Department of Nutrition and Food Safety, Zhejiang Provincial Center for Disease Control and Prevention, Hangzhou, Zhejiang, China; 2 Department of Big Data in Health Science, School of Public Health, Zhejiang University School of Medicine, Hangzhou, China; 3 The Second Affiliated Hospital, Zhejiang University School of Medicine, Hangzhou, China; 4 International School of Medicine, Zhejiang University, Yiwu, Zhejiang, China

**Keywords:** internet use, internet addiction, depression, children, adolescents, China, depressive symptoms

## Abstract

**Background:**

Children’s lives are increasingly mediated by digital technologies, yet evidence regarding the associations between internet use and depression is far from comprehensive and remains unclear.

**Objective:**

This study aimed to investigate the dose-response association between internet use, including use time and addiction behaviors, and depressive symptoms among children and adolescents in Zhejiang Province.

**Methods:**

Data were collected from a school-based health survey China Common Disease and Risk Factor Surveillance Among Students, encompassing 21,336 students in Zhejiang Province. The daily internet use time, internet addiction (IA) behaviors, and depressive symptoms were assessed with questionnaires. Logistic regression models were used to explore the associations of internet use time and IA behaviors with depressive symptoms among children and adolescents. Restricted cubic spline curves were used to determine the dose-response associations.

**Results:**

A total of 6225 (29.2%) students had depressive symptoms. Compared to those reporting no internet use, boys using the internet for >2 hours/day (odds ratio [OR] 1.53, 95% CI 1.34‐1.74) and girls using internet for 1.1‐2 hours/day (OR 1.22, 95% CI 1.06‐1.39) and >2 hours/day (OR 1.70, 95% CI 1.50‐1.93) were at higher risks of depressive symptoms. A significant J-shaped association was identified between internet use time and depressive symptoms among children and adolescents, especially in boys and primary school students (nonlinear *P* values were .006, .003, and <.001, respectively). Increased IA behaviors were associated with a higher odd of depressive symptoms (1 IA behavior: OR 2.01, 95% CI 1.83‐2.21; 2 IA behaviors: 2.91, 95% CI 2.57‐3.29; and ≥3 IA behaviors: 4.72, 95% CI 4.26‐5.22). A positive nonlinear association between the number of IA behaviors and depressive symptoms was found in total population, girls, and primary school students (nonlinear *P* values were .02, .002, .007, respectively).

**Conclusions:**

Findings suggested that excessive internet use time and IA behaviors were significantly associated with an increased risk of depressive symptoms, highlighting the importance of interventions to regulate and educate about adequate internet use during childhood and adolescence.

## Introduction

Depression, characterized by persistent sadness and anhedonia, is a severe mental health disorder that significantly impairs an individual’s functioning and may present with neurovegetative, neurocognitive, and emotional disturbances [1]. The onset of such disorders frequently occurs during childhood or adolescence, with approximately three-quarters of adults with mental disorders receiving their initial diagnosis prior to turning 18 years old [2,3]. Alarmingly, nearly 40% of individuals diagnosed with depression experience their first depressive episode before 20 years of age [4,5]. Globally, depression is prevalent among children and adolescents [6]. In China, this issue has evolved into a significant public health concern, with the prevalence of depression among children and adolescents reaching an estimated 19.8% in 2015 [7]. The implications of depression in childhood extend beyond acute symptoms and carry an increased risk of various adverse outcomes, including suicide attempts [8], substance abuse [9], poor academic performance [10], and impaired social functioning [11]. Additional health impairments, such as poorer self-perceived health [12], obesity [13], and elevated susceptibility to developing anxiety disorder in adulthood, have also been associated with childhood and adolescent depression [14,15]. Therefore, it is imperative to address the risk factors for depression among children and adolescents and develop effective strategies for prevention and intervention [16,17].

In the current digital age, the internet plays a pivotal role in the lives of children and adolescents, presenting both positive and negative impacts on well-being [18,19]. A reasonable duration of internet use can be advantageous [20], yet excessive use and further internet addiction (IA), also known as problematic internet use, may have harmful implications [21]. The World Health Organization (WHO) guidelines recommend limiting time spent being sedentary, especially recreational screen time [22]. According to the Canadian 24-Hour Movement Guidelines, screen time of ≥2 hours/day is considered excessive [23]. IA refers to not only excessive time spent on the internet but also a series of negative symptoms related to behavioral impairment and mental dysfunction [21,24]. The condition of excessive internet use is quite prevalent in youth worldwide [25,26], even among children younger than 5 years [27]. In 2021, China reported approximately 200 million internet users younger than 18 years, contributing to the global user base of around eight billion [18,19]. However, the prevalence of IA among Chinese students is alarmingly high, estimated at 19.5% [19]. This phenomenon raises substantial concerns given the array of adverse outcomes linked to IA, such as obesity [28], elevated perceived stress [29], sleep problems [30,31], diminished quality of life [32], and social isolation [33]. Also, the onset of many mental health disorders is correlated to IA, including attention-deficit/hyperactivity disorder [34], depression, and anxiety [29,35,36]. As such, maintaining appropriate internet use is crucial for children and adolescents to prevent potential harm.

The existing literature offered inconsistent evidence regarding the association between internet use time and depressive symptoms among children and adolescents. While some studies argued for a positive correlation, others suggested that the duration of internet use has a minimal impact on mental health [37-39]. A systematic review indicated that screen-based behaviors were positively associated with depression, while significant associations were only observed in females [18]. In contrast, the positive association between IA and depressive symptoms is more consistently supported [32,40,41]. It is important to note that IA is a condition with varying degrees of severity, and the effects might differ across sexes and developmental stages [42,43].

To address these gaps, this study aimed to investigate the dose-response association between internet use, including time and IA behaviors, and depressive symptoms among children and adolescents in Zhejiang Province, a populous and economically developed province in Eastern China.

## Methods

### Ethical Considerations

Informed consent was obtained from students’ parents or guardians on behalf of the minors. This study protocol was approved by the Ethics Committee of the Zhejiang Provincial Center for Disease Control and Prevention (2020-040-002).

### Study Design and Participants

**Figure 1.**
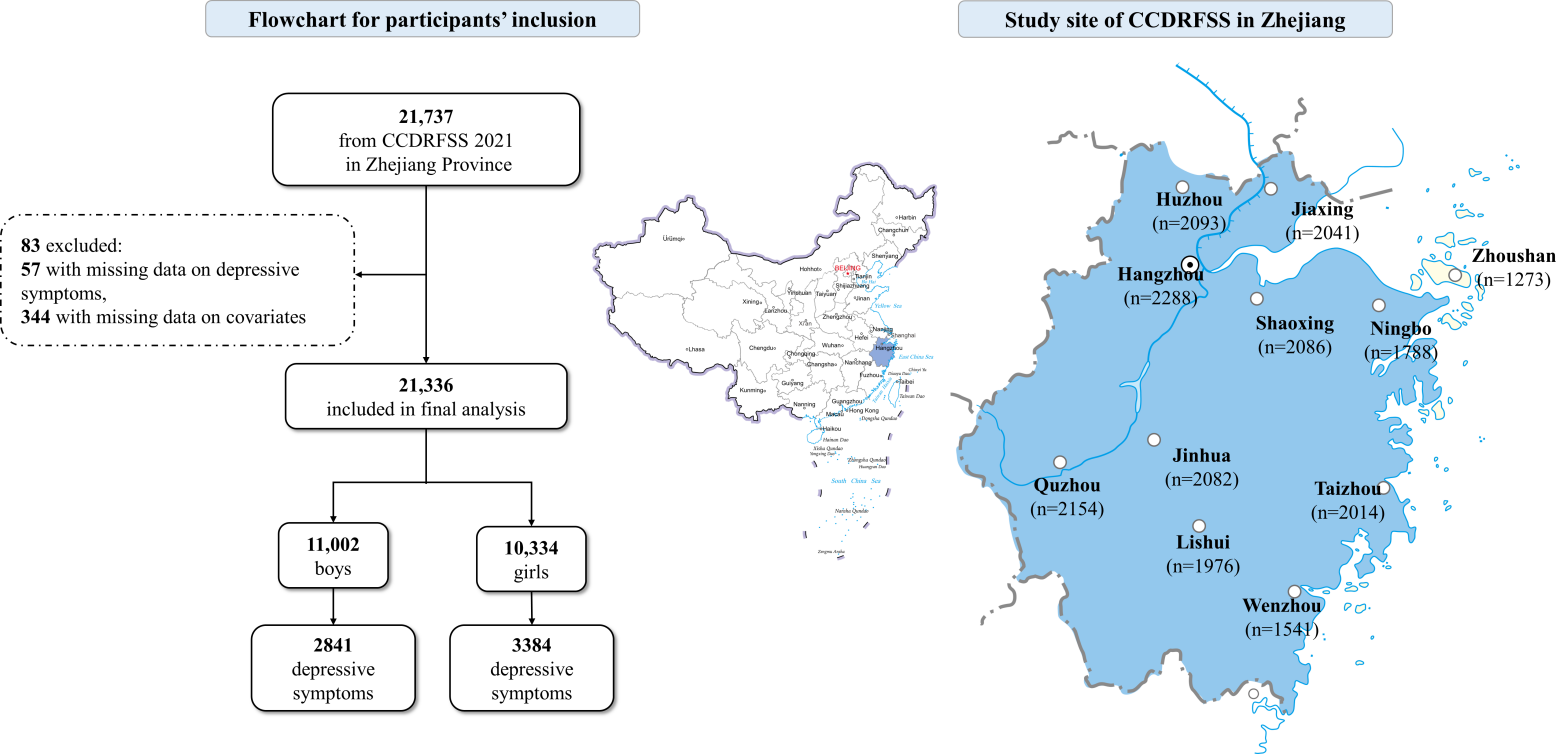
Flowchart of the participants. CCDRFSS: China Common Disease and Risk Factor Surveillance Among Students.

Data for this cross-sectional study were derived from the 2021 China Common Disease and Risk Factor Surveillance Among Students conducted in Zhejiang Province, an annual school-based health survey [44,45]. A stratified cluster sampling was conducted to randomly select one urban area and one rural area from all 11 prefecture-level cities in Zhejiang Province. Schools were then randomly selected across the urban (including two primary schools, two middle schools, two high schools, one vocational high school, and one university) and rural (including two primary school, two middle school, and one high school) regions, covering students from grade 1 and above. To ensure adequate representativeness, we randomly recruited a minimum of 80 students per grade by class units [46,47]. All the students underwent physical examinations implemented by trained health care workers. Students of grade 4 and above were asked to fill out a self-administered questionnaire about their health-related behaviors, and teachers and trained research assistants were present to provide guidance to the students. The initial sample of this study included a total of 21,737 students from grade 4 and above. Furthermore, students with missing data on depressive symptoms (n=57) and covariates (n=344) were excluded, leaving a total of 21,336 students in the final analysis (Figure 1).

### Assessment of Internet Use

The questionnaire asked respondents to report their weekly internet use time and indicate whether they had exhibited specific IA behaviors in the past week. The IA behaviors were assessed using a 10-item IA questionnaire based on the revised version of Young’s diagnostic questionnaire (Cronbach α=0.778) [21,48] (Table S1 in Multimedia Appendix 1). Internet use time was categorized into four groups: 0, 0.1‐1, 1.1‐2, and >2 hours per day. The number of IA behaviors was categorized as follows: 0, 1, 2, or ≥3 [24].

### Assessment of Depressive Symptoms

Depressive symptoms were assessed using the Chinese version of the Center for Epidemiologic Studies Depression Scale, which is a 20-item scale constructed to measure the level of depressive symptoms over the past week [49]. The reliability and validity of the Center for Epidemiologic Studies Depression Scale among the Chinese population have been previously demonstrated [50-52], and the Cronbach α in our study was 0.877. Participants indicated the frequency they encountered each item in the past week on a 4-level scale [53]. Responses ranged from “rarely or none of the time (less than 1 day, 0 point)” to “most or all of the time (5‐7 days, 3 points).” The total score ranged from 0 to 60, with a score of 16 or higher indicating the presence of depressive symptoms [49,54].

### Assessment of Covariates

Demographic information was collected using a validated questionnaire [44,46]. Anthropometric data were measured by trained health staff with standardized equipment. Weight and height were recorded to the nearest 0.1 kg and 0.1 cm, respectively, with participants removing shoes and heavy coats. Covariates adjusted in the study included age, gender (boy or girl), residence (urban or rural), ethnicity (Han or others), number of family members (≤2, 3, 4, or ≥5), grade (primary school: grades 4‐6; middle school: grades 7‐9; or high school; grades 10‐12), boarding (yes or no), BMI (calculated as kg/m^2^), and sedentary time (hour per day).

### Statistical Analysis

In the analysis, all continuous variables exhibited skewness in normality tests and were reported as medians and IQRs. Categorical variables were presented as frequencies and relative percentages (%). To assess differences in characteristics based on the presence or absence of depressive symptoms among students, the Wilcoxon rank sum test was used for continuous variables and the chi-square test was used for categorical variables.

To investigate the association of internet use time (as a categorical variable) and the number of IA behaviors (as a categorical variable) with the risk of depressive symptoms, logistic regression models were performed, and results were presented as the odds ratio (ORs) with 95% CIs. Then, the logistic regression models were stratified by sex and grade. Restricted cubic spline (RCS) curve was used to reveal the dose-response association of internet use time and the number of IA behaviors met with the risk of depressive symptoms. In addition, linear regression models were applied to assess the association of internet use time and IA behaviors with the depressive score, and the dose-response correlation was assessed with 4-knots (obtained at the quartiles of internet use time or number of IA behaviors) RCS curve. All data analyses were performed using STATA 16.0 (StataCorp LLC).

## Results

A total of 21,336 students were included, with a median age of 15.1 (IQR 13.6‐16.6) years. Of these students, 11,002 (51.6%) were boys, and primary school, middle school, and high school students each accounted for 51.2%, 38.3%, and 10.5%. The majority were of Han ethnicity (97.4%), resided in urban areas (62.9%), and were not boarded in school (56.3%). They had a median BMI of 20.3 (IQR 18.4‐23.1) kg/m^2^, a median sedentary time of 7.5 (IQR 3‐10) hours, and reported a median internet use time of 0.3 (IQR 0‐1.2) hours per day. Most students (74.6%) reported no IA behaviors. The median of the depressive score was 12 (IQR 9-16), and 6225 (29.2%) students were identified as having depressive symptoms. Students with depressive symptoms reported significantly longer internet use time and a higher number of IA behaviors compared to their nondepressed peers (*P*<.001; Table 1).

**Table 1.** Characteristics of the included students by depressive status.

Demographics		Total students (N=21,336)	Students without depressive symptoms (n=15,111)	Students with depressive symptoms (n=6225)	*P* value
Age (years), median (IQR)		15.1 (13.6-16.6)	14.8 (13.4-16.4)	15.8 (14.1-17.0)	<.001
**Sex, n (%)**					<.001
	Boy	11,002 (51.6)	8161 (54.0)	2841 (45.6)	
	Girl	10,334 (48.4)	6950 (46.0)	3384 (54.4)	
**Residence, n (%)**					.380
	Urban	13,414 (62.9)	9472 (62.7)	3942 (63.3)	
	Rural	7922 (37.1)	5639 (37.3)	2283 (36.7)	
**Ethnicity, n (%)**					.044
	Han	20,787 (97.4)	14,701 (97.3)	6086 (97.8)	
	Others	549 (2.6)	410 (2.7)	139 (2.2)	
**Number of family members, n (%)**					<.001
	≤2	4657 (21.8)	3158 (20.9)	1499 (24.1)	
	3	6406 (30.1)	4543 (30.1)	1863 (30.0)	
	4	5297 (24.8)	3810 (25.2)	1487 (23.9)	
	≥5	4956 (23.3)	3586 (23.8)	1370 (22.0)	
**Grade, n (%)**					<.001
	Primary school (grades 4‐6)	10,929 (51.2)	8532 (56.5)	2397 (38.5)	
	Middle school (grades 7‐9)	8182 (38.3)	5106 (33.8)	3076 (49.4)	
	High school (grades 10‐12)	2225 (10.5)	1473 (9.7)	752 (12.1)	
**Boarding, n (%)**					<.001
	No	12,011 (56.3)	8797 (58.2)	3214 (51.6)	
	Yes	9325 (43.7)	6314 (41.8)	3011 (48.4)	
BMI (kg/m^2^), median (IQR)		20.3 (18.4-23.1)	20.2 (18.3-23.1)	20.5 (18.6-23.3)	<.001
Sedentary time (hours/day), median (IQR)		7.5 (3.0-10.0)	7.0 (2.3-9.5)	8.5 (5.0-11.3)	<.001
Internet using time (hours/day), median (IQR)		0.3 (0.0-1.2)	0.3 (0.0-1.0)	0.5 (0.0-2.0)	<.001
**Internet use time (hours/day), n (%)**					<.001
	0	8944 (41.9)	6613 (43.8)	2331 (37.4)	
	0.1‐1	6295 (29.5)	4517 (29.9)	1778 (28.6)	
	1.1‐2	2857 (13.4)	2014 (13.3)	843 (13.5)	
	>2	3240 (15.2)	1967 (13.0)	1273 (20.5)	
**Number of internet addictive behaviors met, n (%)**					<.001
	0	15,911 (74.6)	12,330 (81.6)	3581 (57.5)	
	1	2343 (11.0)	1421 (9.4)	922 (14.8)	
	2	1175 (5.5)	599 (4.0)	576 (9.3)	
	≥3	1907 (8.9)	761 (5.0)	1146 (18.4)	

**Figure 2.**
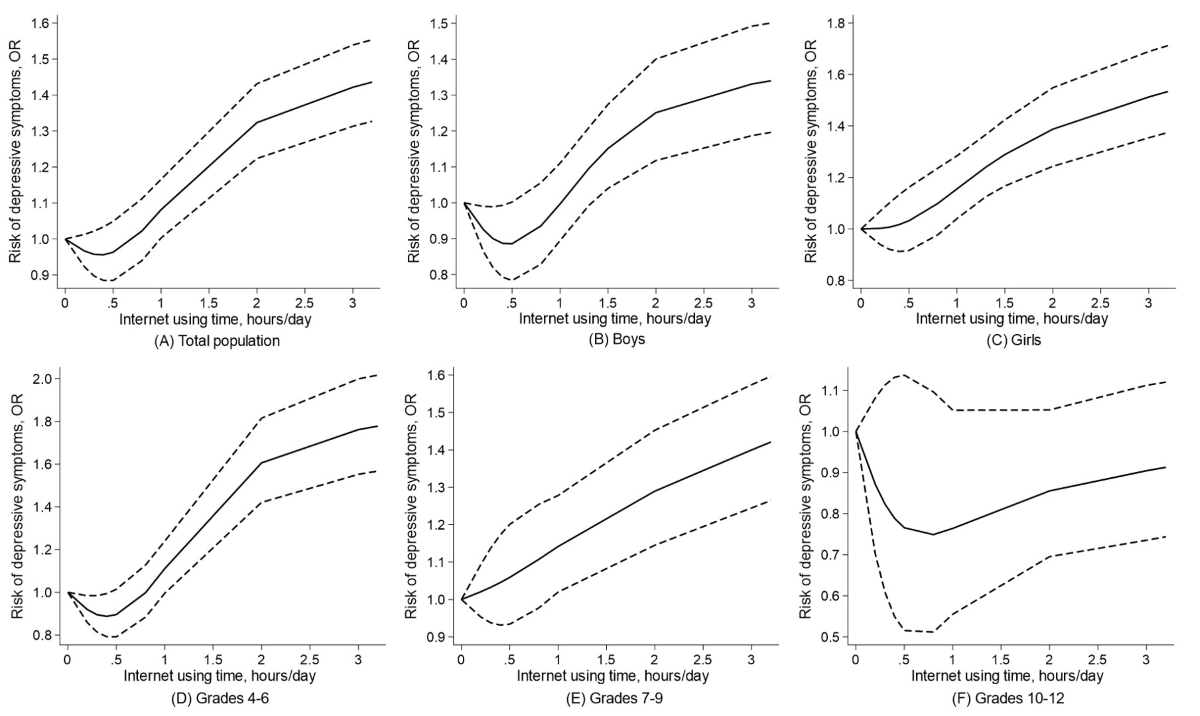
Dose-response association between internet use time and depressive symptoms in total students, by sex and grade The dotted line presents the 95% CI. Models were adjusted for age, sex (except for sex-specific analysis), grade (except for grade-specific analysis), residence, ethnicity, number of family members, boarding, BMI, and sedentary time. *P* nonlinear values were (A) .006 in total population; (B) .003 in boys; (C) .30 in girls; (D) <.001 in grades 4‐6; (E) .85 in grades 7‐9; and (F) .30 in grades 10‐12.

The associations between internet use time and depressive symptoms overall, by sex and grade, are shown in Table 2. When compared with participants reporting no internet use, those with internet use times of 1.1‐2 hours/day and >2 hours/day demonstrated higher risks of depressive symptoms, with ORs of 1.11 (95% CI 1.01‐1.22) and 1.60 (95% CI 1.47‐1.76), respectively. Sex-specific analyses revealed that girls using the internet for 1.1‐2 hours/day and >2 hours/day had elevated risks of depressive symptoms, with ORs of 1.22 (95% CI 1.06‐1.39) and 1.70 (95% CI 1.50‐1.93) respectively, compared to girls reporting no internet use. For boys, only using the internet for >2 hours/day demonstrated a higher risk of depressive symptoms (OR 1.53, 95% CI 1.34‐1.74) compared to those reporting no internet use. Grade-specific analysis demonstrated varied associations across different grades. In grades 4‐6, significant associations were found between internet use time of 1.1‐2 hours/day and >2 hours/day and depressive symptoms (ORs 1.17, 95% CI 1.01‐1.35 and 2.20, 95% CI 1.89‐2.57, respectively). Students in grades 7‐9 showed a less pronounced but still significant association for >2 hours/day (OR 1.60, 95% CI 1.40‐1.84). Interestingly, for students in grades 10‐12, no statistically significant associations were identified. Dose-response analyses revealed a significant J-shaped association between internet use time and depressive symptoms overall among boys and students in grades 4‐6, with nonlinear *P* values of .006, .003, and <.001, respectively (Figure 2). Considering depressive score, for sex stratification, compared to boys without internet use, the boys using the internet for 0.1‐1 hours/day (β=0.40, 95% CI 0.08‐0.72) and >2 hours/day (β=1.31, 95% CI 0.89‐1.73) had elevated risks of depressive scores (Table S2 in Multimedia Appendix 1). For grade stratification, the students from grades 4‐6 using the internet for 0.1‐1 hours/day (β=0.64, 95% CI 0.34‐0.94) and >2 hours/day (β=2.43, 95% CI 1.94‐2.92) had elevated risks of depressive scores. Notably, a minor J-shaped association was found among students from grades 10‐12. Compared to those who reported no internet usage in grade 10‐12, those using the internet for 1.1‐2 hours/day had a decreased risk of depressive scores, with β of −1.23 (95% CI −2.29 to −0.18). No statistically significant associations were identified in the categories of those using the internet for 0.1‐1 hours/day and >2 hours/day in grades 10‐12 (Table S2 in Multimedia Appendix 1). Dose-response associations between internet use time and depressive scores are visualized in Figures S1 and S2 in Multimedia Appendix 1.

**Figure 3.**
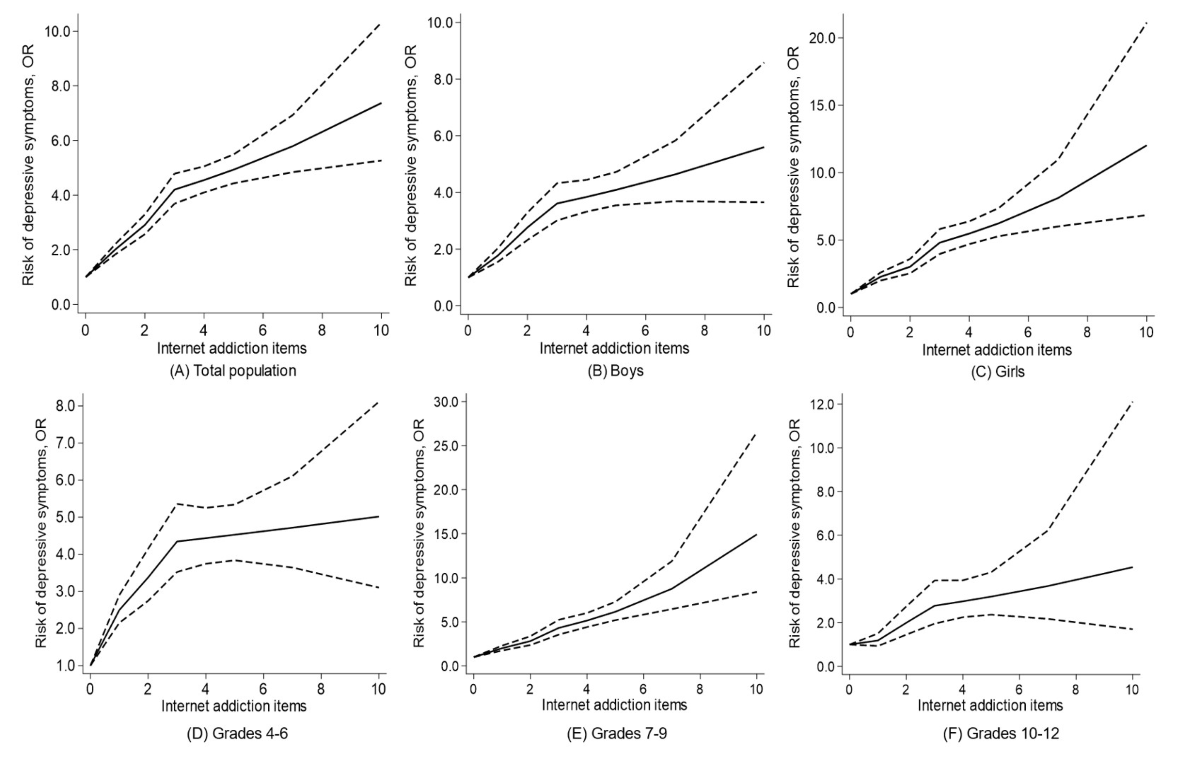
Dose-response association between the number of internet addictive behaviors and depressive symptoms in total students, by sex and grade Notes: The dotted line presents the 95% CI. Models were adjusted for age, sex (except for sex-specific analysis), grade (except for grade-specific analysis), residence, ethnicity, number of family members, boarding, BMI, and sedentary time. *P* nonlinear values were (A) .02 in the total population; (B) .66 in boys; (C) .002 in girls; (D) .007 in grades 4‐6; (E) .06 in grades 7‐9; (F) .24 in grades 10‐12.

Compared with students without IA behaviors, those with 1, 2, and ≥3 IA behaviors exhibited significantly higher risks of depressive symptoms, with ORs of 2.01 (95% CI 1.83-2.21), 2.91 (95% CI 2.57-3.29), and 4.72 (95% CI 4.26-5.22), respectively (Table 2). Sex-stratified analysis showed that boys with 1, 2, and ≥3 IA behaviors had higher risks of depressive symptoms, with ORs of 1.78 (95% CI 1.55‐2.03), 2.77 (95% CI 2.32‐3.30), and 3.98 (95% CI 3.46‐4.58), respectively. Among girls, the risk of depressive symptoms was even higher for those displaying 1, 2, and ≥3 IA behaviors, with ORs of 2.27 (95% CI 1.99‐2.59), 3.01 (95% CI 2.52‐3.60), and 5.69 (95% CI 4.90‐6.62), respectively. When stratified by grade, students in grades 4‐6 with 1, 2, and ≥3 IA behaviors had increased risks of depressive symptoms, with ORs of 2.50 (95% CI 2.15‐2.90), 3.38 (95% CI 2.75‐4.15), and 4.49 (95% CI 3.82‐5.28), respectively. Students in grades 7‐9 had corresponding ORs of 2.00 (95% CI 1.74‐2.30), 2.82 (95% CI 2.36‐3.36), and 5.45 (95% CI 4.68‐6.35), respectively, for 1, 2, and ≥3 IA behaviors, respectively. Among students in grades 10‐12, the ORs were 1.18 (95% CI 0.93‐1.50), 1.99 (95% CI 1.45‐2.74), and 3.04 (95% CI 2.31‐4.00), respectively, for 1, 2, and ≥3 IA behaviors. A dose-response association between increasing numbers of IA behaviors and higher risks of depressive symptoms was found (Figure 3). Meanwhile, the elevated trend slowed down in both sexes and all grades for ≥3 IA behaviors. Similar results were found in the associations between IA and depressive score in total students, by sex and grade (Table S2 and Figures S3 and S4 in Multimedia Appendix 1).

**Table 2.** Association between internet use and depressive symptoms in total students, by sex and grade. Models were adjusted for age, sex (except for sex-specific analysis), grade (except for grade-specific analysis), residence, ethnicity, number of family members, boarding, BMI, and sedentary time. *P<.05, **P<.001.

		Total students (N=21,336)	Sex		Grade		
			Boys (n=11,002)	Girls (n=10,334)	Grades 4‐6 (n=10,929)	Grades 7‐9 (n=8182)	Grades 10‐12 (n=2225)
**Internet use time (hours/day), OR^a^ (95% CI)**							
	0	Reference	Reference	Reference	Reference	Reference	Reference
	0.1‐1	1.04 (0.97-1.13)	1.01 (0.91-1.12)	1.08 (0.97-1.20)	1.04 (0.94-1.16)	1.09 (0.98-1.22)	0.92 (0.63-1.35)
	1.1‐2	1.11 (1.01-1.22)*	1.00 (0.87-1.15)	1.22 (1.06-1.39)*	1.17 (1.01-1.35)*	1.13 (0.98-1.30)	0.79 (0.59-1.05)
	>2	1.60 (1.47-1.76)**	1.53 (1.34-1.74)**	1.70 (1.50-1.93)**	2.20 (1.89-2.57)**	1.60 (1.40-1.84)**	1.01 (0.83-1.24)
**Number of internet addictive behaviors met, OR (95% CI)**							
	0	Reference	Reference	Reference	Reference	Reference	Reference
	1	2.01 (1.83-2.21)**	1.78 (1.55-2.03)**	2.27 (1.99-2.59)**	2.50 (2.15-2.90)**	2.00 (1.74-2.30)**	1.18 (0.93-1.50)
	2	2.91 (2.57-3.29)**	2.77 (2.32-3.30)**	3.01 (2.52-3.60)**	3.38 (2.75-4.15)**	2.82 (2.36-3.36)**	1.99 (1.45-2.74)**
	≥3	4.72 (4.26-5.22)**	3.98 (3.46-4.58)**	5.69 (4.90-6.62)**	4.49 (3.82-5.28)**	5.45 (4.68-6.35)**	3.04 (2.31-4.00)**

a OR: odds ratio.

## Discussion

### Principal Findings

In our study, longer internet use time was associated with a higher risk of depressive symptoms among Chinese children and adolescents overall, for both sexes, and among primary and middle school students. Interestingly, in grades 10‐12, no significant association between internet use time and depressive symptoms was observed. A notable J-shaped association between internet use and depressive symptoms was observed overall among boys and students in grades 4‐6. For IA, a larger number of IA behaviors was associated with a higher risk of depressive symptoms, with a significant nonlinear association in total students, girls, and students in grades 4‐6. Moreover, all participants but high school students with longer internet use time presented higher depressive scores. For IA behaviors, a higher number was associated with a higher depressive score in both sexes and all grades.

Our findings added to the existing body of research on the impact of internet use on mental health but with some differences. Previous studies have reported a positive association between heavy internet use and depressive symptoms [55,56]. The observed J-shaped association also highlighted the possibility of a protective effect associated with a minimal amount of internet use (around 0.5 h/day). This could be explained by the positive aspects of internet use, such as social connection, access to support networks, and educational resources [57,58]. While excessive use could lead to isolation, sleep disturbances, and exposure to harmful content, thereby increasing the risk of depression [59-61]. The displacement theory indicates that heavy internet use would reduce children’s intimacy with their family members as well as social involvement, which may lead to stressful interpersonal relationships and increase psychological problems [43]. The sex-specific differences in the associations between internet use time and depressive symptoms could be due to the different ways in which boys and girls use the internet, or the difference in how they respond to online experiences. For instance, girls might be more affected by social comparisons or cyberbullying, which are common on online platforms [62,63]. For the diminished association between internet use time and depressive symptoms among senior grades, one explanation for this could be the maturation and development of coping strategies in older adolescents, enabling them to handle the stresses associated with the internet more effectively [64]. Another plausible explanation might be that older students are using the internet for more educational purposes, which may not have the same impact on depressive symptoms as recreational use [65].

Our study provides compelling evidence that a higher number of IA behaviors are significantly associated with a higher risk of depressive symptoms among students. These findings align with previous research suggesting a strong link between IA and depressive symptoms. For instance, a systematic review by Lopes et al [37] found a significant association between IA and depression. Our study adds to this body of evidence by demonstrating a nonlinear increase in the risk of depressive symptoms with an increasing number of IA behaviors, which could be due to the increasing disruption of normal life activities, social isolation, and exposure to negative online experiences with higher levels of IA [66]. The increase in depressive risks was more pronounced in the early stage (≤3) of IA behaviors, which underscored the importance of early control of excessive internet use and IA symptoms. Similar to the findings for the associations between internet use time and depressive symptoms, the observed sex differences between IA and depressive symptoms might also be due to the vulnerability of girls in the online activities, such as social comparisons or cyberbullying [67]. The varying impact of IA behaviors across different school grades could also be explained by the resilience and coping mechanisms among students in different developmental stages, where senior students might have developed better digital literacy skills and coping strategies to manage the potential negative effects of IA. To mitigate the negative impact of excessive internet use, parents should attach importance to creating a positive home media environment, as well as involving their children in alternative activities like reading and exercising [31].

### Strengths and Limitations

There are several strengths in our study. First, we used the RCS curve to show the nonlinear dose-response association. This approach provides a better fit to the data and reduces the degrees of freedom, enhancing the accuracy, reliability, and precision of our study. Second, the database used in this study was derived from a large representative sample of children and adolescents in Zhejiang Province, China. Moreover, sex- and grade-specific subgroup analyses were performed separately, capturing the variations in the association between internet use and depressive symptoms.

However, our study is not without limitations. The cross-sectional design precluded our ability to establish causality. A lack of detailed information regarding the age of IA initiation and family environment factors, all of which could potentially influence adolescent depressive symptoms, limited the depth of our analysis. Furthermore, our participant sample was drawn exclusively from an economically developed region of China. This may limit the extent to which our findings are representative of the broader population of Chinese children and adolescents.

### Conclusions

A significant association between internet use and depressive symptoms was demonstrated, with excessive internet use and more IA behaviors being associated with higher risks of depressive symptoms. A notable J-shaped association between internet use time and depressive symptoms was observed among boys and primary school students. Early preventive and mitigating interventions aimed at promoting appropriate internet use should be prioritized to prevent depressive symptoms among children and adolescents. Teachers and parents should help children develop healthy internet use habits and monitor children’s internet activities actively. Future studies could explore the longitudinal associations between internet use at different developmental stages and subsequent mental disorders in youth and investigate potential sex differences.

## Supplementary material

10.2196/53101 Multimedia Appendix 1 Supplementary tables.

## Abbreviations

IA: internet addiction
OR: odds ratio
RCS: restricted cubic spline
