# Supplementary material for: Dose-Response Associations of Internet Use Time and Internet Addiction With Depressive Symptoms Among Chinese Children and Adolescents: Cross-Sectional Study
Source: JMIR Public Health Surveill. 2024 Sep 23;10:e53101. doi: 10.2196/53101 (PMC11423272; doi:10.2196/53101)
Supplement: Multimedia Appendix 1 [file publichealth-v10-e53101-s001.docx]

Legends

[Table S1. Items of the internet addiction questionnaire 2](#_Toc142033321)

[Table S2. Association between internet use and depressive score in total students, by sex and grade 3](#_Toc142033322)

[Figure S1. Dose-response association between internet using time and depressive scores in total population and by sex 5](#_Toc142033323)

[Figure S2. Dose-response association between internet using time and depressive scores by grade 6](#_Toc142033324)

[Figure S3. Dose-response association between internet addiction and depressive scores in total population and by sex 7](#_Toc142033325)

[Figure S4. Dose-response association between internet addiction and depressive scores by grade 8](#_Toc142033326)

| **Item** | **Statement** | **Option** |
| --- | --- | --- |
| 1 | Internet using time exceed 4 hours in a day | yes/no |
| 2 | Feel preoccupied with the Internet (keep thinking about previous online activity while not using the Internet) | yes/no |
| 3 | Feel restless or unwilling to do anything else when stopping Internet use and get eased when using Internet | yes/no |
| 4 | Use Internet with increasing amount of time in order to achieve satisfaction | yes/no |
| 5 | Lose interest in other recreational activities (hobbies, meeting friends, etc.) because of the internet | yes/no |
| 6 | Repeatedly made unsuccessful efforts to control, cut back or stop Internet use | yes/no |
| 7 | Unable to complete homework or skip school because of the Internet | yes/no |
| 8 | Lie to parents, teachers or schoolmates to conceal the extent of involvement with the Internet | yes/no |
| 9 | Continue to use the Internet despite knowing the negative consequences (e.g. lack of sleep, being late for school, and arguing with parents) | yes/no |
| 10 | Use the Internet as a way of escaping from the reality or problems, or of relieving feelings of helplessness, anxiety or depression | yes/no |

# Table S1. Items of the internet addiction questionnaire

# Table S2. Association between internet use and depressive score in total students, by sex and grade

|  |  | **Total students (N=21336)** | **Sex** | | **Grade** | | |
| --- | --- | --- | --- | --- | --- | --- | --- |
|  |  |  | **Boys  (N=11002)** | **Girls (N=10334)** | **Grade 4-6 (N=10929)** | **Grade 7-9 (N=8182)** | **Grade 10-12 (N=2225)** |
|  |  | ***β* (95% CI)** | | | | | |
| **Internet use time, hours/day** | | |  |  |  |  |  |
|  | 0 | ref | ref | ref | ref | ref | ref |
|  | 0.1-1.0 | **0.54 (0.31, 0.77)** | **0.40 (0.08, 0.72)** | **0.67 (0.33, 1.01)** | **0.64 (0.34, 0.94)** | **0.57 (0.20, 0.94)** | 0.08 (-1.32, 1.48) |
|  | 1.1-2.0 | **0.33 (0.03, 0.64)** | -0.18 (-0.60, 0.24) | **0.83 (0.40, 1.27)** | 0.18 (-0.24, 0.59) | **0.82 (0.35, 1.29)** | **-1.23 (-2.29, -0.18)** |
|  | >2.0 | **1.64 (1.34, 1.93)** | **1.31 (0.89, 1.73)** | **1.99 (1.56, 2.41)** | **2.43 (1.94, 2.92)** | **1.69 (1.23, 2.16)** | 0.12 (-0.64, 0.87) |
| **Numbers of internet addiction met** | | |  |  |  |  |  |
|  | 0 | ref | ref | ref | ref | ref | ref |
|  | 1 | **2.15 (1.85, 2.45)** | **1.66 (1.23, 2.08)** | **2.66 (2.23, 3.09)** | **2.56 (2.10, 3.03)** | **2.30 (1.85, 2.75)** | 0.54 (-0.32, 1.39) |
|  | 2 | **3.79 (3.38, 4.20)** | **3.50 (2.92, 4.08)** | **4.05 (3.47, 4.63)** | **4.37 (3.69, 5.04)** | **3.61 (3.04, 4.19)** | **2.44 (1.23, 3.64)** |
|  | ≥3 | **5.84 (5.51, 6.17)** | **4.79 (4.33, 5.26)** | **6.93 (6.46, 7.40)** | **5.70 (5.18, 6.23)** | **6.11 (5.65, 6.57)** | **4.72 (3.68, 5.76)** |

Notes: CI, confidence interval. Models were adjusted for age, sex (not for sex-specific analysis), grade, residence, ethnicity, number of family members, living in school, body mass index, sedentary time.

# Figure S1. Dose-response association between internet using time and depressive scores in total population and by sex

Notes: Models were adjusted for age, grade, residence, ethnicity, number of family members, boarding, body mass index, and sedentary time. *P* nonlinear for total population, boys and girls were 0.602, 0.684, and 0.227, respectively.

# Figure S2. Dose-response association between internet using time and depressive scores by grade

Notes: Models were adjusted for age, sex, residence, ethnicity, number of family members, boarding, body mass index, and sedentary time. *P* nonlinear for grades 4-6, grades 7-9, and grades 10-12 were 0.877, 0.319, and 0.241, respectively.

# Figure S3. Dose-response association between internet addiction and depressive scores in total population and by sex

Notes: Models were adjusted for age, grade, residence, ethnicity, number of family members, boarding, body mass index, and sedentary time. *P* nonlinear for total population, boys and girls were 0.406, 0.454, and 0.042, respectively.

# Figure S4. Dose-response association between internet addiction and depressive scores by grade

Notes: Models were adjusted for age, sex, residence, ethnicity, number of family members, boarding, body mass index, and sedentary time. *P* nonlinear for grades 4-6, grades 7-9, and grades 10-12 were 0.516, 0.129, and 0.227, respectively.
